# Comparative risk perceptions of switching to JUUL vs. continued smoking and subsequent switching away from cigarettes: a longitudinal observational study

**DOI:** 10.1186/s40359-023-01351-8

**Published:** 2023-10-05

**Authors:** Arielle Selya, Saul Shiffman

**Affiliations:** Pinney Associates, Inc, 201 N Craig St., Suite 320, Pittsburgh, PA 15213 USA

**Keywords:** Cigarettes, Electronic nicotine Delivery Systems, JUUL, Risk perceptions, Smoking, Switching

## Abstract

**Background:**

Evidence indicates that electronic nicotine delivery systems (ENDS) pose lower risk than cigarettes; however, many smokers harbor misperceptions that ENDS are equally or *more* harmful, possibly deterring them from switching. This study examines whether comparative risk perceptions of JUUL vs. smoking are associated with subsequent switching, among smokers who recently purchased JUUL.

**Methods:**

*N* = 16,996 current established smokers who recently purchased a JUUL Starter Kit were followed 6 times over 12 months. Comparative risk perceptions were assessed using both direct and indirect measures (i.e., contrasting JUUL and smoking directly in questions, and deriving from separate absolute scales). Repeated-measures logistic regression examined switching across follow-up (no smoking in past 30 days) as a function of baseline risk perceptions, adjusting for demographics and baseline smoking behavior.

**Results:**

Perceiving JUUL as less harmful than smoking was associated with higher switching rates, using both direct (e.g., adjusted odds ratio [AOR] = 1.48 for “JUUL much less” vs. “more/much more harmful”) and indirect (AOR = 1.07, for each 10-unit increase in fraction; AOR = 1.51 for highest (6-100) vs. lowest (0 to < 1) fraction categories) comparative risk measures (all *p* < 0.0001). Among the subset smoking 10 + cigarettes per day, associations between risk perceptions and switching were more pronounced (AOR = 2.51 for “JUUL much less” vs. “more/much more harmful”; AOR = 1.81 for 6-100 vs. 0 to < 1 fraction, both *p* < 0.0001).

**Conclusions:**

Smokers who perceive JUUL as less harmful than cigarettes have higher odds of switching. Future research should examine whether messaging which aligns comparative risk perceptions with current evidence can facilitate switching, especially among heavier smokers.

## Background

Electronic nicotine delivery systems (ENDS) are alternative nicotine-delivery products that electronically heat a nicotine-containing solution into aerosol, which avoids the harmful emissions due to combustion inherent in cigarette smoking. Reviewers have concluded that ENDS pose a small fraction of the risk compared to combustible cigarettes [1, 2], and thus ENDS have substantial potential for harm reduction [3] among adult smokers who would not otherwise quit smoking in the near term. However, tobacco harm reduction has been controversial, both out of concern for youth use and dependence [4] and because ENDS are not risk-free [2, 3, 5]. For example, 9.4% of youth used ENDS in the past 30 days in 2022 [6], and concerns have been raised about frequent ENDS use [6] and nicotine dependence in youth [7]. Regarding ENDS use among adult smokers, concerns have been raised about cumulative harmful exposures among dual users of ENDS and cigarettes [8], although for many, dual use is a transitional stage to smoking cessation or switching [9, 10] and often accompanied by substantial reductions in cigarette smoking [10, 11]. A substantial proportion of adult ENDS users also report some degree of nicotine dependence and difficulty quitting ENDS [12], and continued nicotine dependence is a concern for smokers who switch to ENDS [13]; however, dependence on ENDS is generally lower than cigarette dependence [14]. These risks and benefits of ENDS must be weighed carefully.

Despite ENDS’ lower risk profile compared to cigarettes, the majority of US smokers hold misperceptions that ENDS are equally or *more* harmful than cigarette smoking [15], likely due, in part, to misinformation in the public sphere that overemphasizes the risks of ENDS use [16, 17]. (Note that use of wording such as ‘holding misperceptions,’ is *not* meant to imply that the individuals in question are in any way to blame for their misunderstandings.) These risk misperceptions (i.e., risk perceptions that are not supported by current evidence) are becoming more common over time [18], consistent with recent incidents of misinformation about the health risks of ENDS in public discourse. Risk perceptions of ENDS and cigarettes may be important in influencing the use of these substances according to behavioral theories [19]. For example, subjective expected utility models [20], the theory of planned behavior [21], and the health belief model [22] all posit that perceived risk or benefit of a behavior are central in adopting that behavior [21, 23]: people are more likely to adopt a behavior change that they perceive as having a benefit. The importance of risk perceptions has been demonstrated in a wide range of health behaviors, including vaccination [24], COVID protective measures [25], and driving behavior [26], in addition to tobacco use [27–29]. As a result, current misperceptions that ENDS are equally or more harmful than cigarettes may pose a barrier for smokers to switch completely away from cigarettes to ENDS, thus undermining ENDS’ potential for harm reduction [18, 30].

Risk perceptions could impact several stages of switching among a range of smokers, from those who have never tried ENDS to those who have successfully switched using ENDS at least once. In a longitudinal analysis of the nationally-representative Population Assessment of Tobacco and Health (PATH) study, Kim et al. [27] examined the prospective relationship between comparative risk perceptions and subsequent ENDS-related behaviors across three milestones of ENDS use and smoking. First, ENDS-naïve smokers who perceived ENDS as having less risk than smoking were more likely to subsequently initiate ENDS. Other studies have reported similar findings [28–30]. Second, those who perceived ENDS as having lower risk than smoking were more likely to subsequently switch away from smoking with ENDS (also, [31]). Finally, even among adult smokers who had already switched in the past year, Kim et al. reported that those who perceived ENDS as equally risky to smoking were more likely to resume smoking.

However, less is known about how risk perceptions *at the time smokers initiate a new round of ENDS use* relate to later switching, in contrast to previous literature which assesses risk perceptions at some (varying) time before or after ENDS initiation [31] – which only captures switching from that point on, often omitting switching that occurred prior to the survey. Thus, assessing risk perceptions at the time of ENDS initiation, and subsequent switching, provides more complete capture of switching behavior among all smokers who tried ENDS. Additionally, prior studies do not always consider the temporal order of risk perceptions and ENDS use, raising the possibility that smokers who use ENDS may have adapted their risk perceptions to existing behavior, rather than vice versa. Thus, assessing risk perceptions at the point of initiating a particular ENDS ensures the appropriate temporal sequence for examining whether risk perceptions may impact tobacco use patterns. Finally, focusing on a single ENDS brand eliminates heterogeneity across different ENDS products, possibly allowing clearer focus on the association between risk perceptions and switching. A rich source of data for this question is the previously-described Adult JUUL Switching and Smoking Trajectories (ADJUSST) study [32] which enrolled a large national sample of adult established smokers who had first purchased a JUUL brand starter kit, and followed them over the subsequent year. Previous research on the ADJUSST study showed that, among established smokers, lighter baseline smoking behavior, more frequent JUUL use, and greater satisfaction with and dependence on JUUL were positively associated with later switching [33]. Risk perceptions have not yet been examined in the ADJUSST study.

The current secondary analysis of the above ADJUSST study [32] focuses on risk perception and subsequent switching, among adult established smokers who had recently adopted JUUL brand ENDS with the purchase of a JUUL Starter Kit. We hypothesize that smokers who perceive JUUL to be less harmful than cigarettes are more likely to later switch away from smoking, consistent with psychological theory and prior research [31, 34]. We also hypothesize that these findings will be robust across different ways of assessing risk perceptions [35–37]: namely, both “direct comparative risk,” where the comparison between JUUL and smoking is explicit within the question (e.g. “is using JUUL less/equally/more harmful than smoking cigarettes?”) and “indirect comparative risk,” where a measure of perceived risk is derived from comparing participants’ responses to separate items (e.g. “how likely are you to develop lung cancer from using JUUL” and “… from smoking cigarettes”).

## Methods

### Sample

Data were drawn from the Adult JUUL Switching and Smoking Trajectories (ADJUSST) Study, which has been described in detail previously [32]. Briefly, *N* = 22,905 adult (21+) current established smokers were selected from the full ADJUSST sample (i.e. smoked 100 + cigarettes lifetime, and were now smoking “some days” or “every day”) who purchased a JUUL Starter Kit online or in a retail store in 2018 and were followed up to 6 times over 12 months (at 1, 2, 3, 6, 9 and 12 months after baseline). A previous study reported that 23.4% of this sample used ENDS other than JUUL regularly at baseline [38]. Participants completed the survey online, and were compensated $30 USD for each survey. Importantly, this was a non-interventional naturalistic observational study of JUUL use and smoking, and participants did not set explicit goals for switching or quitting in the context of this study, and they were not provided with goals, counseling, or products. The ADJUSST study was approved by the Advarra® Institutional Review Board, Federalwise Assurance number (FWA) 00023875.

The current analyses focus on those who completed at least one follow-up (*N* = 17,986). From these, participants were excluded whose reported account of risk perceptions were illogical, which is indicative of a response bias such as random or inattentive responding that increases noise in the data. Because validity checks to identify inattentive responding were not built into ADJUSST, identifying and removing participants who provide contradictory responses on these similar items is one strategy to reduce this source of measurement error. Specifically, participants were excluded if they purchased the JUUL System for the reasons that “I believed JUUL might be less harmful to me than cigarettes” or “I had read/saw information on the internet about the health benefits of switching from smoking cigarettes to using e-cigarettes”, but then stated they perceived JUUL to be *more* risky than smoking, according to either direct or indirect comparative risk measures described below (*N* = 762). Participants whose direct and indirect risk perceptions were inconsistent with each other (i.e. on one measure, responded that JUUL was riskier than smoking, and on the other, responded in the category with lowest comparative JUUL risk, see variable definitions below) were also excluded (a further *N* = 25). Finally, participants who, despite purchasing a JUUL Starter Kit, never reported using their JUUL were additionally excluded (i.e., those who reported not using their JUUL in 30 days prior to each completed follow-up, *N* = 141), as our aim was to study participants who switched using JUUL (vs. other methods). The final analytic sample was *N* = 17,058.

### Measures

#### Baseline comparative risk perceptions of smoking vs. JUUL

*Direct* comparative risk perceptions were assessed with a single item which directly compared smoking and JUUL use: “In your opinion, is using the JUUL device less harmful, about the same, or more harmful than smoking cigarettes?” with responses on a 5-point scale ranging from “much less harmful” to “much more harmful,” as has been used previously [39, 40]. Due to low endorsement of these response categories, the “more harmful” and “much more harmful” groups were combined, consistent with other studies [39, 41].

*Indirect* comparative risk perceptions were derived from two sets of four items each on perceived *absolute* risk of adverse health outcomes from smoking (lung cancer, lung disease other than lung cancer (e.g. chronic obstructive pulmonary disease, emphysema), heart disease, and early/premature death), assessed separately for continued smoking at participant’s current rate, and for switching completely to JUUL, respectively. Participants indicated what they thought their chances are of having each of the health outcomes at some point during their lifetime, on a 0-100% scale (101 possible response values). The 4 items for continued smoking and for switching to JUUL, respectively, were averaged into one score for each, as factor analysis indicated that each scale was unidimensional (first factor accounted for 84.7% and 88.6% of the variance, respectively) and resulted in high internal consistency reliability (Cronbach’s alpha = 0.94 and 0.96, respectively). Baseline cigarettes per day (CPD) was significantly correlated with smoking risk scores (Spearman’s rho = 0.31, *p* < 0.0001), providing evidence for convergent validity of this score.

Using these two scores, indirect comparative risk was expressed as the fraction of perceived absolute risk for smoking vs. risk of switching to JUUL (i.e. how many times more harmful continued smoking is, compared to switching to JUUL). A fraction measure was used because it is more sensitive to smaller *relative* differences at low values of *absolute* risk, which was expected for light smokers, given the explicit anchoring of the smoking risk items in respondents’ current smoking levels. To avoid division by zero, values of 0 risk for JUUL, which were uncommon (8.8%), were recoded as 1. The fraction was top-coded at 100 (indicating the perception that smoking is 100 times as risky as JUUL), thus capping the variable at the highest value (i.e., greatest risk reduction) that has strong empirical support (i.e., evidence that ENDS that have 1% or less of the emissions of conventional cigarettes [42–44]), while lower values of this variable represent a range of perceptions (from more modest perceived risk reductions, to perceived equivalent risk, to perceived increased risk).

The indirect risk fraction was analyzed both as a continuous variable (main analyses) and as a categorical variable (follow-up analyses), grouping scores into 5 levels: ( 1) below 1 (referred to as “0 to < 1”, i.e., switching to JUUL is riskier than continued smoking; (2) 1 to < 2 (i.e., switching to JUUL is equally to half as risky as continued smoking); (3) 2 to < 3 (i.e., switching to JUUL is one-third as risky as continued smoking); (4) 3 to < 6; and (5) 6 to 100.

#### Switching away from cigarettes

Switching away from cigarettes was defined as no smoking in the past 30 days (“in the past 30 days, have you smoked a cigarette, even one or two puffs?”), consistent with previous literature [45, 46]. Of those who switched (e.g., ~ 51% at 12 months), the majority were using JUUL only (~ 46%), though some (~ 5%) were using neither JUUL nor cigarettes, as previously reported [10]. All non-missing data were used across follow-ups, consistent with previous studies [32, 47].

#### Baseline smoking behavior

Baseline smoking behavior was measured with three separate variables: (1) daily-average CPD across both smoking- and nonsmoking-days in the past 30 days, accounting for both frequency of smoking (i.e., number of days smoked in the past 30 days) and quantity on days smoked (i.e., number of cigarettes), (2) number of days smoked in the past 30 days, and (3) smoking duration in years.

#### Demographic characteristics

Demographic characteristics included age in years, sex, race/ethnicity; education; annual income, and employment.

### Analyses

The association between comparative risk at baseline and switching away from smoking at follow-up was examined using repeated-measures logistic regression models (generalized estimating equations (GEE)), using all available data across follow-ups and statistically controlling for interdependence among observations contributed by the same individuals. Participants’ demographics and baseline smoking behavior (CPD, number of days smoked in the past 30, and years of smoking duration) were adjusted for in these models. Average adjusted switching rates were calculated as the average of projected outcomes among participants in each risk perception category, based on the adjusted GEE model and calculated at the mean (for continuous variables) or most frequent category (for categorical variables) of all other covariates.

Direct and indirect risk measures were analyzed in separate models. Direct comparative risk was analyzed as an ordinal variable, both comparing switching rates against the reference group of participants with the highest perceived risk of JUUL (presented in the tables), and across each adjacent pair of categories, since the categories are ordinal (presented in the figures). Indirect comparative risk fraction was analyzed both as a continuous variable (examining both linear and quadratic terms) and a 5-level categorical variable to further examine the association (again using a reference-group comparison in the tables, and a comparison between adjacent pairs of categories in the figures).

Because the indirect comparative risk items explicitly reference participants’ *own* current levels of smoking, it is possible that risk perceptions vary by baseline smoking heaviness. Characteristics of participants who smoked 10 or more CPD (*N* = 8,047) were compared with those who smoked < 10 CPD at baseline, and the above repeated-measures logistic regression analyses were conducted on the subset who smoked 10 + CPD at baseline, who were expected to have an appreciable level of perceived smoking risk.

## Results

Characteristics of the full sample are shown in Table 1. These adult established smokers smoked 9 CPD on average, most smoked daily in the past 30 days, and had been smoking for 10 years on average. Average *absolute* risk perceptions of smoking were high (average perceived risk of 4 health conditions at participants’ own smoking levels was 63.8%), and absolute risk perceptions of JUUL were low (20.0%), resulting in most established smokers perceiving JUUL to be less harmful than continued smoking, according to both direct (89.9% perceived JUUL to be less or much less harmful) and indirect measures (participants perceived smoking as 2.6 times as harmful as JUUL, on average).

**Table 1:** Baseline characteristics of the full sample and by baseline CPD (< 10 vs. 10+)

**Baseline Characteristic**		**Full Sample (*N=*** **17,058)**	**<10 CPD Smokers** **(*N=*** **8,504)**	**10+ CPD Smokers** **(*N=*** **8,047)**
Age, mean (SD)		32.8 (10.8)	30.1 (9.5)	35.6 (11.3)
Sex	Male	54.6% (*N=*9244)	55.8% (*N=*4718)	53.9% (*N=*4309)
	Female	44.9% (*N=*7611)	43.5% (*N=*3681)	45.8% (*N=*3667)
	Transgender	0.5% (*N=*89)	0.7% (*N=*63)	0.3% (*N=*23)
Race/ethnicity	Non-Hisp White	78.6% (*N=*12,554)	72.3% (*N=*5778)	85.1% (*N=*6413)
	Non-Hisp Black	2.9% (*N=*457)	3.6% (*N=*290)	2.0% (*N=*153)
	Non-Hisp Asian	5.6% (*N=*902)	7.8% (*N=*622)	3.5% (*N=*264)
	Hispanic	8.4% (*N=*1342)	11.2% (*N=*895)	5.5% (*N=*413)
	Other/multi	4.5% (*N=*721)	5.1% (*N=*406)	3.9% (*N=*296)
Education	High school or less	27.4% (*N=*4378)	22.6% (*N=*1804)	31.9% (*N=*2412)
	Some college/AA	43.5% (*N=*6960)	41.7% (*N=*3325)	45.8% (*N=*3465)
	Bachelor’s or more	29.2% (*N=*4666)	35.8% (*N=*2854)	22.4% (*N=*1693)
Income	<$50k	52.8% (*N=*7830)	52.8% (*N=*3910)	52.5% (*N=*3686)
	$50-$100k	29.8% (*N=*4427)	28.6% (*N=*2118)	31.2% (*N=*2187)
	>$100k	17.4% (*N=*2581)	18.7% (*N=*1384)	16.3% (*N=*1146)
Employment	Don’t work for pay	12.6% (*N=*2033)	11.4% (*N=*920)	13.8% (*N=*1054)
	< 15 hrs/week	2.5% (*N=*408)	3.1% (*N=*248)	1.9% (*N=*148)
	15-34 hrs/week	11.6% (*N=*1869)	13.4% (*N=*1082)	9.6% (*N=*732)
	35+ hrs/week	73.3% (*N=*11,833)	72.0% (*N=*579)	74.6% (*N=*5692)
Average CPD over P30D, median (IQR)		9.0 (2.7-15.0)	2.7 (1.0-5.0)	15.0 (11.6-20.0)
Smoking days in P30D, median (IQR)		30.0 (20.0-30.0)	20.0 (9.0-28.0)	30.0 (30.0-30.0)
Smoking duration in years, median (IQR)		10.0 (4.0-19.0)	6.0 (3.0-12.0)	15.0 (8.0-23.0)
Smoking risk score^a^, median (IQR)		63.8 (48.8-87.5)	52.5 (33.8-77.5)	75.0 (50.0-92.5)
JUUL risk score^b^, median (IQR)		20.0 (10.0-40.0)	18.8 (7.5-34.8)	25.0 (10.0-45.0)
Indirect comparative risk fraction^c^, median (IQR)		2.6 (1.7-5.0)	2.5 (1.6-5.0)	2.7 (1.7-5.3)
Direct comparative risk category^d^				
JUUL more/much more harmful:		0.4% (*N=*67)	0.5% (*N=*38)	0.3% (*N=*25)
About the same:		9.7% (*N=*1565)	10.5% (*N=*849)	8.9% (*N=*672)
JUUL less harmful:		62.9% (*N=*10,098)	64.0% (*N=*5167)	61.8% (*N=*4656)
JUUL much less harmful:		27.0% (*N=*4330)	25.0% (*N=*2020)	29.0% (*N=*2183)

Note: *CPD* Cigarettes per day, *IQR* Interquartile range, *P30D* Past 30 days, *SD* standard deviation

^a^Average of 4 items: chance (0-100%) of having lung cancer, other lung disease, heart disease, or premature death at some point if continue to smoke at current rate

^b^Average of 4 items: chance (0-100%) of having lung cancer, other lung disease, heart disease, or premature death at some point if switch completely to JUUL

^c^Smoking risk score/JUUL risk score. Values < 1 indicate perceptions that JUUL is more harmful than smoking; values > 1 indicate perceptions that smoking is more harmful than JUUL

^d^“In your opinion, is using the JUUL device less harmful, about the same, or more harmful than smoking cigarettes?” Assessed on a 5-point scale ranging from “much less harmful” to “much more harmful.”

### Association between comparative risk perceptions and subsequent switching among all baseline established smokers

**Fig. 1:**
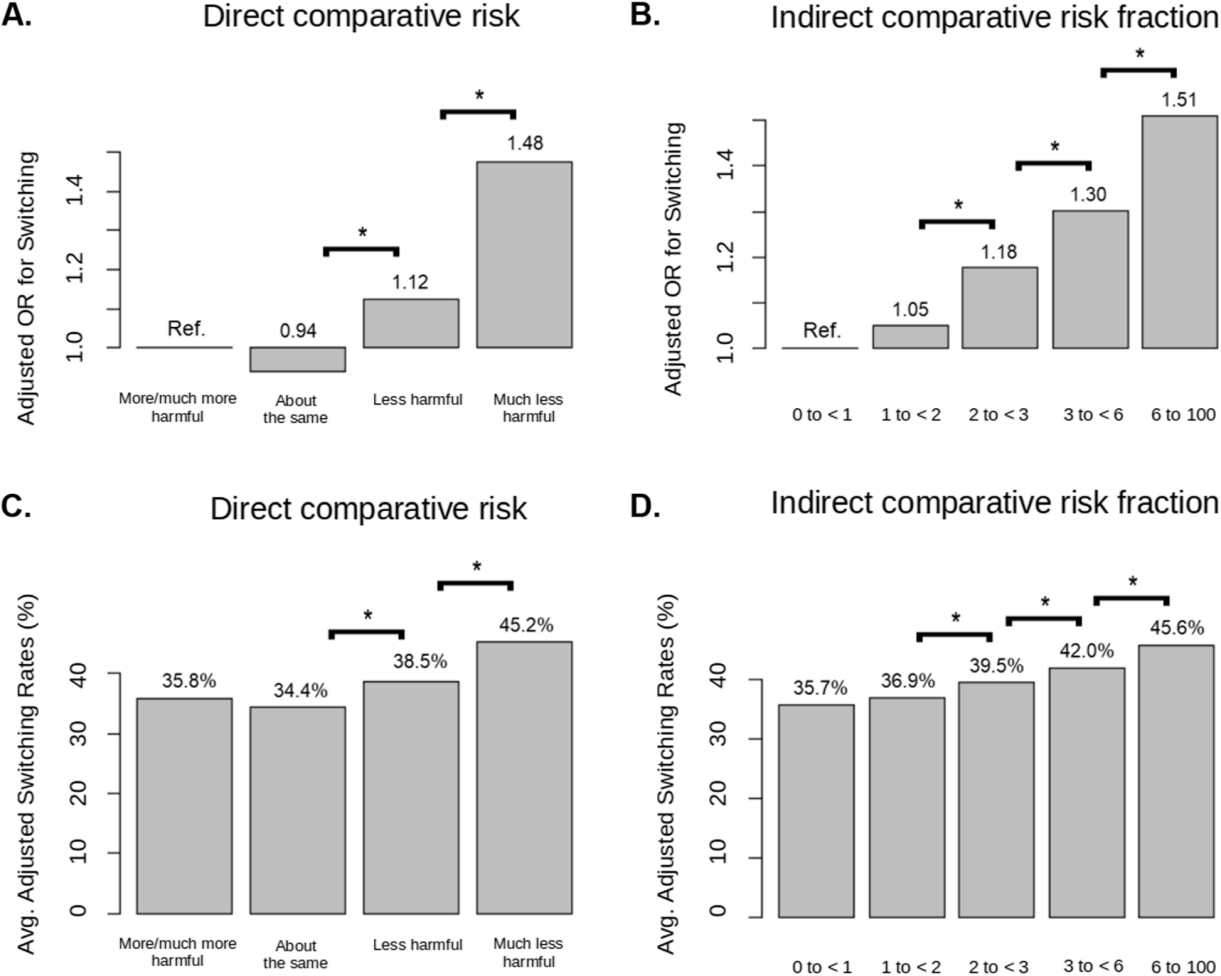
AORs and switching rates by comparative risk perception groups, among all baseline established smokers. **A** and **B**: Adjusted odds ratios (AORs) for switching (y-axis) for risk perception groups (x-axis), for both direct (**A**) and indirect (**B**) comparative risk perceptions. **C** and **D**: Average adjusted switching rates across direct (**C**) and indirect (**D**) risk perception groups. Analyses adjust for baseline smoking history and demographic characteristic (see text for details) (see text for details). Brackets and * indicate significance at *p*<.05 between adjacent categories based on GEE model (see text). Indirect comparative risk fraction = 1 indicates equal perceived risk of switching to JUUL and continued smoking, and is included in the “=1 to <2” group. Indirect risk fraction <1 (“=0 to <1” group) indicates that continued smoking is perceived to be less risky than switching to JUUL; values > 1 indicate that switching to JUUL is perceived to be less risky than continued smoking

In the full sample, *direct* comparative risk perceptions of JUUL being comparatively less risky than smoking were significantly associated with higher odds of switching, after adjusting for baseline smoking behavior and demographic characteristics (Fig. 1A, which uses the same reference group for all risk perception categories; and Table 2, which presents sequential contrasts between risk perception groups). Those who perceived JUUL as “much less harmful” than smoking had the highest odds of switching, followed by those who perceived JUUL as “less harmful,” which in turn was significantly higher than those who perceived JUUL as “about the same” or “more/much more harmful” (the latter two did not significantly differ). Those who perceived JUUL as “much less harmful” had the highest covariate-adjusted switching rate based on this model, at 45.2%, more than a third higher than the 35.8% of those who perceived JUUL as “more/much more harmful” (Fig. 1C).

**Table 2:** Adjusted odds ratios of subsequent switching across baseline risk perceptions, among all baseline established smokers

Predictor		AOR (95% CI) of Switching, Model 1 (direct)	AOR (95% CI) of Switching, Model 2 (indirect, continuous)	AOR (95% CI) of Switching, Model 3 (indirect, categorical)
Direct comparative risk	JUUL more/much more harmful	Ref.	--	--
	About the same (vs. more/much more harmful)	0.94 (0.71 – 1.24), *p *= 0.662		
	JUUL less harmful (vs. about the same)	**1.20 (1.12 – 1.27),** ***p*** **< 0.001**		
	JUUL much less harmful (vs. less harmful)	**1.31 (1.26 – 1.37),** ***p*** **< 0.001**		
Indirect comparative risk (continuous)	Linear term	--	**1.007 (1.006 – 1.008),** ***p*** **< 0.001**	--
	Quadratic term		**0.9999 (0.9998 – 0.9999),** ***p*** **< 0.001**	
Indirect comparative risk (categorical)	0 to <1	--	--	Ref.
	1 to <2 (vs. 0 to <1)			1.05 (0.90 – 1.23), *p *= 0.521
	2 to <3 (vs 1 to <2)			**1.12 (1.06 – 1.17),** ***p*** **< 0.001**
	3 to <6 (vs 2 to <3)			**1.11 (1.05 – 1.17),** ***p*** **< 0.001**
	6 to 100 (vs 3 to <6)			**1.16 (1.10 – 1.22),** ***p*** **< 0.001**
Sex	Male	Ref.	Ref.	Ref.
	Female	1.00 (0.96 – 1.03), *p *= 0.833	**0.96 (0.93 – 1.00),** ***p*** **= 0.031**	**0.96 (0.93 – 1.00),** ***p*** **= 0.032**
	Transgender	0.80 (0.64 – 1.00), *p *= 0.055	0.81 (0.64 – 1.02), *p *= 0.077	0.81 (0.64 – 1.03), *p *= 0.081
Race/ethnicity	Non-Hisp white	Ref.	Ref.	Ref.
	Non-Hisp black	1.08 (0.97 – 1.20), *p *= 0.158	1.10 (0.99 – 1.22), *p *= 0.087	1.11 (1.00 – 1.24), *p *= 0.051
	Non-Hisp Asian	**0.88 (0.81 – 0.95),** ***p*** **= 0.001**	**0.86 (0.80 – 0.94),** ***p*** **< 0.001**	**0.87 (0.80 – 0.94),** ***p*** **< 0.001**
	Hispanic	0.97 (0.91 – 1.04), *p *= 0.392	0.95 (0.89 – 1.01), *p *= 0.095	0.96 (0.90 – 1.02), *p *= 0.181
	Other/multi	0.95 (0.87 – 1.03), *p *= 0.228	0.95 (0.88 – 1.04), *p *= 0.266	0.96 (0.88 – 1.04), *p *= 0.345
Age		1.00 (1.00 – 1.00), *p *= 0.853	1.00 (1.00 – 1.01), *p *= 0.086	1.00 (1.00 – 1.01), *p *= 0.181
Education	High school or less	Ref.	Ref.	Ref.
	Some college/AA	**0.88 (0.85 – 0.92),** ***p*** **< 0.001**	**0.90 (0.87 – 0.94),** ***p*** **< 0.001**	**0.90 (0.86 – 0.94),** ***p*** **< 0.001**
	Bachelor’s or more	**0.82 (0.78 – 0.86),** ***p*** **< 0.001**	**0.83 (0.79 – 0.87),** ***p*** **< 0.001**	**0.82 (0.78 – 0.86),** ***p*** **< 0.001**
Income	<$50k	Ref.	Ref.	Ref.
	$50-$100k	**1.07 (1.02 – 1.11),** ***p*** **= 0.003**	**1.07 (1.03 – 1.12),** ***p =*** **0.001**	**1.07 (1.02 – 1.11),** ***p*** **= 0.002**
	>$100k	**1.16 (1.10 – 1.23),** ***p*** **< 0.001**	**1.15 (1.09 – 1.21),** ***p*** **< 0.001**	**1.14 (1.08 – 1.20),** ***p*** **< 0.001**
Employment	Don’t work for pay	Ref.	Ref.	Ref.
	< 15 hrs/week	**0.84 (0.74 – 0.95)** ***p*** **= 0.006**	**0.81 (0.72 – 0.92),** ***p =*** **0.001**	**0.81 (0.71 – 0.91),** ***p*** **= 0.001**
	15-34 hrs/week	0.95 (0.89 – 1.02), *p *= 0.191	0.95 (0.88 – 1.02), *p *= 0.147	0.94 (0.88 – 1.01), *p *= 0.101
	35+ hrs/week	**1.08 (1.02 – 1.14),** ***p*** **= 0.007**	1.07 (1.01 – 1.13), *p *= 0.016	**1.07 (1.01 – 1.13),** ***p*** **= 0.024**
Average CPD over P30D		**0.98 (0.98 – 0.99),** ***p*** **< 0.001**	**0.98 (0.98 – 0.99),** ***p*** **< 0.001**	0.98 (0.98 – 0.99), ***p*** **< 0.001**
Smoking days in P30D		**0.98 (0.97 – 0.98),** ***p*** **< 0.001**	**0.98 (0.97 – 0.98),** ***p*** **< 0.001**	0.98 (0.97 – 0.98), ***p*** **< 0.001**
Smoking duration in years		**0.98 (0.98 – 0.98),** ***p*** **< 0.001**	**0.98 (0.98 – 0.98),** ***p*** **< 0.001**	0.98 (0.98 – 0.98), ***p*** **< 0.001**

Note: Separate GEE models were run using each measure of comparative risk perceptions (Model 1: Direct comparative risk, Model 2: Continuous indirect risk fraction; Model 3: Categorical indirect risk fraction), adjusting for all covariates listed in table (see text for details)

*AOR *Adjusted odds ratio, *CPD *Cigarettes per day, *GEE *Generalized estimating equation, *IQR *Interquartile range, *P30D *Past 30 days

Similarly, among the entire sample of established smokers, *indirect* perceived risk fractions were modestly but significantly associated with subsequent switching, such that for every 10-unit increase in the risk fraction of continued smoking compared to switching to JUUL, the odds of switching across the follow-up period increased by 7% (adjusted odds ratio [AOR] = 1.07, 95% confidence interval [CI]: 1.06–1.08, *p* < 0.001). This linear association was modified by a small negative quadratic term (AOR = 0.999, CI = 0.998–0.999, *p* < 0.001), indicating that the increase in switching slowed slightly at higher levels of the indirect risk fraction.

This curvilinear relationship is evident in Fig. 1, panel B using a 5-level categorized version of the indirect comparative risk fraction (Fig. 1B). Those who perceived JUUL as increasingly less risky compared to smoking had successively higher switch rates, with the exception that switching rates were similar between those who viewed JUUL as riskier than cigarettes (indirect risk fraction < 1) and those who viewed JUUL as equal to, or up to half the risk of, smoking (indirect risk fraction between 1 and < 2). Those who perceived the risk of JUUL to be 1/6th or less that of smoking had the highest adjusted switching rate (45.6%, Fig. 1D), again more than a third higher than the 35.7% of those who perceived JUUL to be riskier than smoking.

### Association between comparative risk perceptions and subsequent switching among baseline established smokers smoking at least 10 cigarettes per day

Characteristics of those who smoked 10 + CPD, compared to those who smoked < 10 CPD at baseline, are shown in Table 1. The subset who smoked 10 + CPD at baseline, as expected, on average smoked more frequently (30 vs. 20 days in the past 30 days) and had a longer smoking duration (15 vs. 6 years) than those who smoked < 10 CPD. Also as expected based on the wording of indirect comparative risk items (i.e., with reference to one’s own current levels of smoking), the 10 + CPD smokers perceived substantially greater absolute risk from smoking (75 vs. 52.5), and to a much lesser degree, greater absolute risk from switching to JUUL (25 vs. 19). Together, this amounted to 10 + CPD smokers expecting comparatively more reduction in risk when switching to JUUL, which is observed in both direct and indirect measures.

**Fig. 2:**
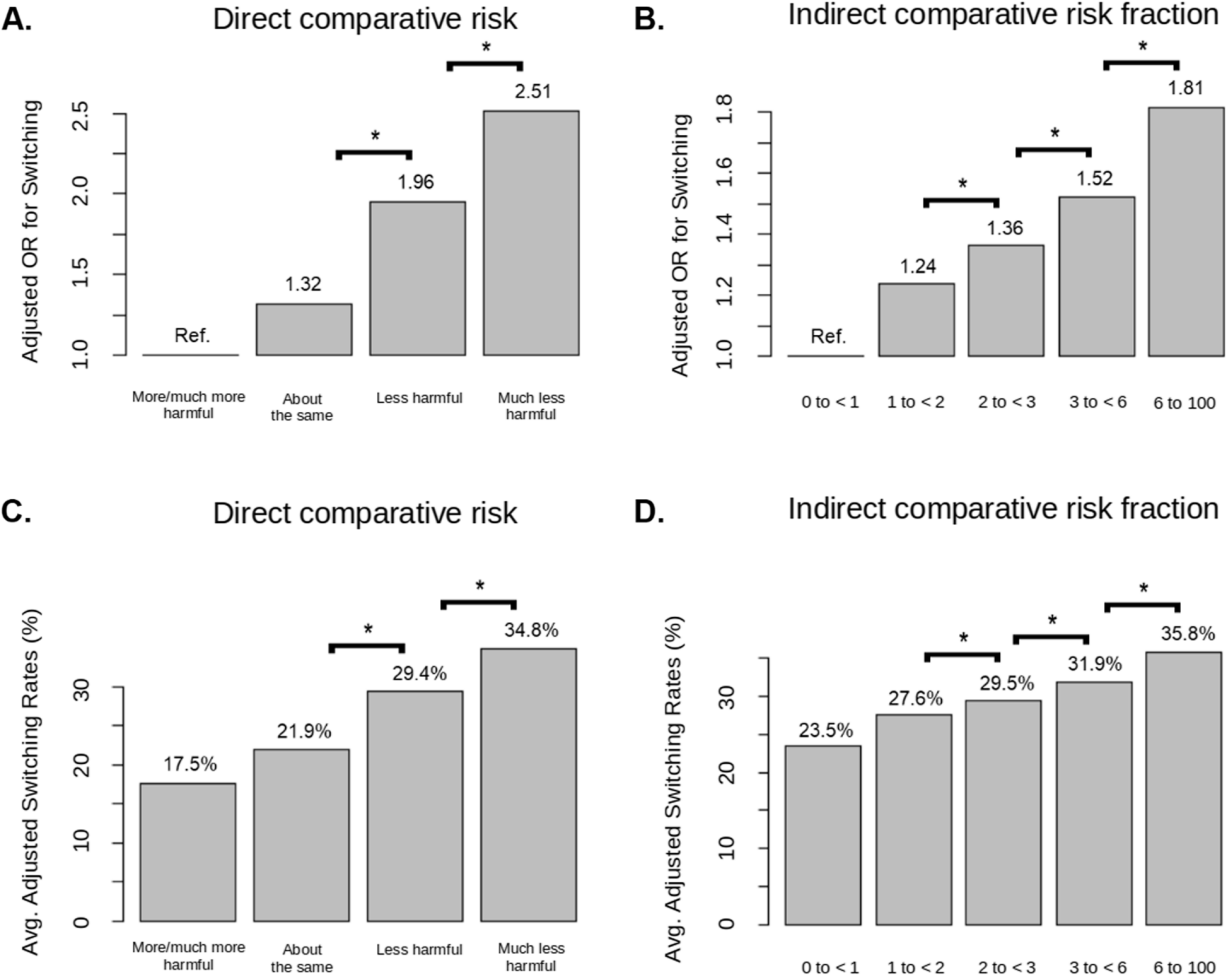
AORs and switching rates by comparative risk perception groups, among baseline established smokers with 10+CPD. **A** and **B**: Adjusted odds ratios (AORs) for switching (y-axis) for risk perception groups (x-axis), for both direct (**A**) and indirect (**B**) comparative risk perceptions. **C** and **D**: Average adjusted switching rates across direct (**C**) and indirect (**D**) risk perception groups. Analyses adjust for baseline smoking history and demographic characteristic (see text for details) (see text for details). Brackets and * indicate significance at p<.05 between adjacent categories based on GEE model (see text). Indirect comparative risk fraction = 1 indicates equal perceived risk of switching to JUUL and continued smoking, and is included in the “=1 to <2” group. Indirect risk fraction <1 (“=0 to <1” group) indicates that continued smoking is perceived to be less risky than switching to JUUL; values > 1 indicate that switching to JUUL is perceived to be less risky than continued smoking

 In the subset who smoked 10 + CPD at baseline, the association between baseline risk perceptions and subsequent switching was more pronounced and more clearly monotonic (Fig. 2A; Table 3), with respect to both direct and indirect risk perceptions. For *direct* risk perceptions, those who perceived greater reduction in JUUL compared to smoking had successively higher odds of switching, with the exception that those who perceived JUUL as “about the same” or “more/much more harmful” had similar switching rates. Average adjusted switching rates also monotonically increased across risk perception groups, with the highest switching rates (34.8%) among those who perceive JUUL to be “much less harmful” than cigarettes (Fig. 2C), almost double the 17.5% switching rate in those who perceived JUUL to be “more/much more harmful.”

**Table 3:** Adjusted odds ratios of subsequent switching across baseline risk perceptions, among 10 + CPD smokers

Predictor		AOR (95% CI) of Switching, Model 1	AOR (95% CI) of Switching, Model 2	AOR (95% CI) of Switching, Model 3
Direct comparative risk	JUUL more/much more harmful	Ref.		
	About the same (vs. more/much more harmful)	1.32 (0.76–2.30), *p* = 0.323		
	JUUL less harmful (vs. about the same)	**1.48 (1.34–1.63)**, ***p*** **< 0.001**		
	JUUL much less harmful (vs. less harmful)	**1.29 (1.21–1.36)**, ***p*** **< 0.001**		
Indirect comparative risk (continuous)	Linear term		**1.007 (1.005–1.008)**, ***p*** **< 0.001**	
	Quadratic term		**0.9999 (0.9998–0.9999)** ***p*** **< 0.001**	
Indirect comparative risk (categorical)	0 to < 1			Ref.
	1 to < 2 (vs. 0 to < 1)			1.24 (0.94–1.63), *p* = 0.124
	2 to < 3 (vs. 1 to < 2)			**1.10 (1.02–1.18)**, ***p*** **= 0.011**
	3 to < 6 (vs. 2 to < 3)			**1.12 (1.04–1.21)**, ***p*** **= 0.004**
	6 to 100 (vs. 3 to < 6)			**1.19 (1.11–1.28)**, ***p*** **< 0.001**
Sex	Male	Ref.	Ref.	Ref.
	Female	1.01 (0.96–1.07), *p* = 0.632	0.97 (0.92–1.03) *p* = 0.285	0.97 (0.92–1.02), *p* = 0.276
	Transgender	0.63 (0.38–1.06), *p* = 0.085	0.62 (0.36–1.06), *p* = 0.082	0.63 (0.38–1.07), *p* = 0.089
Race/ethnicity	Non-Hisp white	Ref.	Ref.	Ref.
	Non-Hisp black	**1.26 (1.05–1.52)**, ***p*** **= 0.014**	**1.32 (1.10–1.59)**, ***p*** **= 0.003**	**1.35 (1.12–1.62)**, ***p*** **= 0.001**
	Non-Hisp Asian	0.91 (0.79–1.06), *p* = 0.228	0.93 (0.81–1.08), *p* = 0.366	0.94 (0.82–1.09), *p* = 0.426
	Hispanic	0.99 (0.88–1.11), *p* = 0.815	0.94 (0.84–1.06), *p* = 0.314	0.95 (0.85–1.06), *p* = 0.373
	Other/multi	0.98 (0.86–1.12), *p* = 0.771	1.02 (0.89–1.16), *p* = 0.787	1.03 (0.90–1.18), *p* = 0.632
Age		**0.99 (0.99–1.00)**, ***p*** **= 0.003**	1.00 (0.99–1.00), *p* = 0.185	1.00 (0.99–1.00), *p* = 0.150
Education	High school or less	Ref.	Ref.	Ref.
	Some college/AA	**0.86 (0.81–0.91)**, ***p*** **< 0.001**	**0.88 (0.83–0.94)**, ***p*** **< 0.001**	**0.87 (0.82–0.93)**, ***p*** **< 0.001**
	Bachelor’s or more	**0.83 (0.77–0.90)**, ***p*** **< 0.001**	**0.84 (0.78–0.90)**, ***p*** **< 0.001**	**0.83 (0.77–0.89)**, ***p*** **< 0.001**
Income	<$50k	Ref.	Ref.	Ref.
	$50-$100k	**1.14 (1.07–1.21)**, ***p*** **< 0.001**	**1.17 (1.10–1.24)**, ***p*** **< 0.001**	**1.16 (1.09–1.24)**, ***p*** **< 0.001**
	>$100k	**1.30 (1.20–1.41)**, ***p*** **< 0.001**	**1.31 (1.21–1.42)**, ***p*** **< 0.001**	**1.29 (1.19–1.40)** ***p*** **< 0.001**
Employment	Don’t work for pay	Ref.	Ref.	Ref.
	< 15 h/week	0.95 (0.78–1.17), *p* = 0.656	0.91 (0.74–1.11), *p* = 0.352	0.89 (0.73–1.09), *p* = 0.259
	15–34 h/week	0.90 (0.81–1.01), *p* = 0.069	**0.87 (0.78–0.98)**, ***p*** **= 0.018**	**0.87 (0.77–0.97)**, ***p*** **= 0.011**
	35 + hrs/week	1.03 (0.95–1.11), *p* = 0.534	1.02 (0.94–1.10), *p* = 0.698	1.01 (0.93–1.09), *p* = 0.802
Average CPD over P30D		**0.99 (0.98–0.99)**, ***p*** **< 0.001**	**0.99 (0.99–0.99)**, ***p*** **< 0.001**	**0.99 (0.99–0.99)**, ***p*** **< 0.001**
Smoking days in P30D		**0.95 (0.94–0.96)**, ***p*** **< 0.001**	**0.95 (0.94–0.96)**, ***p*** **< 0.001**	**0.95 (0.93–0.96)**, ***p*** **< 0.001**
Smoking duration in years		**0.98 (0.98–0.99)**, ***p*** **< 0.001**	**0.98 (0.98–0.99)**, ***p*** **< 0.001**	**0.98 (0.98–0.99)**, ***p*** **< 0.001**

Note: Separate GEE models were run using each measure of comparative risk perceptions (Model 1: Direct comparative risk, Model 2: Continuous indirect risk fraction; Model 3: Categorical indirect risk fraction), adjusting for all covariates listed in table (see text for details)

*AOR *Adjusted odds ratio, *CPD *Cigarettes per day, *GEE *Generalized estimating equation, *IQR *Interquartile range, *P30D *Past 30 days

Among the subset who smoked 10 + CPD at baseline, more positive *indirect* comparative risk perceptions were also associated with higher switching rates, such that for every 10-unit increase in the indirect risk fraction of continued smoking compared to switching to JUUL, the odds of switching increased by 7% (AOR: 1.07, 95% CI: 1.05–1.08, *p* < 0.001). This linear trend was modified by a small negative quadratic term (AOR = 0.999, CI: 0.998–0.999, *p* < 0.001).

The association between indirect comparative risk fraction and subsequent switching is shown in Fig. 2B, using the 5-level categorized expression of the indirect comparative risk fraction. Those who perceived JUUL to be comparatively less risky than smoking had successively higher odds of switching, again with the exception that switching rates were similar between those who perceived JUUL to be riskier than smoking, and those who perceived JUUL to be equal to smoking or half as risky. Switching rates also increased monotonically across groups with larger indirect risk fractions, with an adjusted switching rate of 35.8% for those who perceive JUUL to be 1/6th as risky as smoking (Fig. 2D), which is approximately one-third higher than the 23.5% switching rate for those who with indirect risk fraction < 1.

## Discussion

The current analyses found that among established smokers who had already purchased a JUUL Starter Kit, those who perceived JUUL to be less harmful compared to smoking were significantly more likely to switch completely away from smoking over the year following that purchase. This finding was consistent across both direct and indirect measures of comparative risk perceptions, and among all established smokers as well as those smoking at least 10 cigarettes a day.

Switching rates varied along only one end of the comparative risk perception continuum: among those who perceived at least *some* reduction in risk from switching to JUUL, those who perceived comparatively larger risk reductions had successively higher switching rates. On the other hand, among those who harbored misperceptions about JUUL’s risk compared to smoking (who again are not to blame for these misperceptions), there was no difference between those who perceived JUUL to be *more* harmful than smoking and those who perceived the two to be (approximately) *equally* harmful. If risk perceptions are causally related to switching behavior, as suggested by behavioral theories [21, 23], this implies that moving smokers’ risk perceptions of JUUL, or other ENDS, from “more harmful” to “equally harmful” may not improve switching rates. The data suggest that maximum switching rates may not be achieved unless smokers’ comparative risk perceptions fully reflect the *magnitude* of current best estimates of risk reduction. For example, aligning smokers’ risk perceptions with converging estimates across the literature that ENDS are at least 95% safer than cigarettes [2, 43, 44, 48] would correspond to an indirect comparative risk fraction of 20 or higher, putting them in the group with the highest odds of switching. Corrective messaging would still be important even if ENDS had a more modest reduction in risk compared to cigarettes: even aligning smokers’ risk perceptions to an overestimation of ENDS’ risk – i.e. that that ENDS have 1/3rd the risk of cigarettes [49], which is now acknowledged as an overestimate [50] – would move most smokers from the comparative risk perception groups with the lowest switching rates (i.e., an indirect risk fraction of 0–2), to the group with the second-highest switching rates (i.e., an indirect risk fraction of 3). Future research is needed to examine whether messaging or education on the comparative exposures and, by implication, risks of ENDS vs. cigarettes, can correct harmful misperceptions about ENDS. The importance of accurate communication is recognized by the US Food & Drug Administration’s Center for Tobacco Products, which has recently highlighted the need to educate adult smokers about the relative risk of different tobacco products, especially e-cigarettes [51].

An additional consideration is that we use the term “switching” to refer both to participants who switched away from smoking and used only JUUL at follow-up, as well as those who discontinued use of both products, consistent with previous literature [1, 47]. This definition is also consistent with studies of nicotine replacement therapy (NRT) which attribute cessation to NRT even if NRT is no longer being used [52]. As described above, the current analytic sample was limited to respondents who used JUUL during at least one follow-up, and JUUL-only use was likely an intermediate stage for those who eventually discontinued use of both products: as previously reported, increases in JUUL-only use approximately offset declines in dual use over time [10]. Thus, all participants in the current analytic sample who switched used JUUL in at least part of their transition away from cigarettes.

Accounting for baseline cigarette consumption was important in understanding the relationship between comparative risk perceptions and subsequent switching. The role of CPD in this association is complex: on one hand, smokers with higher CPD are *less* likely to switch [33], but on the other hand, these heavier smokers perceive greater risk reduction in switching to JUUL, which makes them *more* likely to switch.

The subgroup analysis of 10 + CPD smokers confirms the importance of baseline smoking behavior, as the relationship between comparative risk perceptions and switching was more pronounced and monotonic among this heavier-smoking subgroup. Though differences in switching rates remained non-significant among the two most unfavorable risk perception groups, the effect sizes were larger and showed more distinct differences among the 10 + CPD subgroup than among the full sample.

Although the *direct* comparative risk item did not explicitly reference participants’ own smoking levels, it showed a similar relationship with participants’ own smoking rate, and with subsequent switching. This suggests that in their judgment of the risk of smoking, smokers may implicitly be responding with their own cigarette consumption levels in mind, even when they were not explicitly directed to do so. This may be due to the questions on direct risk perceptions being asked first, possibly resulting in participants carrying this hypothetical condition into the later questions used in absolute comparative risk.

Given that a majority of adults incorrectly perceive ENDS to be as or more harmful than smoking [15, 18], these misperceptions may present a barrier for adult smokers in switching from cigarettes to ENDS, thus perpetuating the high public health burden from combustible tobacco. This finding has important implications for facilitating switching among adult smokers based on correcting misperceptions that they harbor. Previous research on reduced-risk messages shows that exposure to such information lowers adults’ perceived risk of ENDS and increases their intentions to use ENDS [27–29]. Similar findings have been reported for correcting smokers’ misperceptions about the risk of nicotine replacement therapy [53]. These findings suggest that messaging that highlights the comparative risk differential of ENDS compared to cigarettes could promote realistically favorable risk perceptions, which in turn could increase switching away from cigarettes. Though it is important to note that the current findings are specific to a group of smokers who already purchased JUUL – and thus who likely already perceived ENDS as less harmful – other research supports the importance of risk perceptions and therefore corrective messaging in other groups of smokers as well, including those who had not previously used ENDS [34, 54], and those with more unfavorable risk perceptions, who stand to benefit from larger corrections in their risk perceptions. Notably, the Center for Tobacco Products at FDA has announced plans for a public information campaign to correct risk misperceptions [51].

### Limitations

The current observational study cannot determine whether the association between risk perceptions and switching is causal. Nevertheless, risk perceptions temporally precede switching in these data, consistent with causality; however, this analysis is limited to correlational relationship between the two. The generalizability of findings may also be limited. The sample is not nationally representative, though it is large and national. Further, the current findings may not generalize to non-JUUL ENDS, although previous research found an association between risk perceptions of ENDS in general and switching [31]. Additionally, since the current study focused on smokers who already initiated JUUL use, the range of risk perceptions is likely skewed towards perceiving JUUL as less harmful for this sample, compared to smokers more generally; this suggests that the overall association between comparative risk perceptions and switching is likely stronger than observed here. The ADJUSST study did not assess data on detailed use patterns of other ENDS, or use of vaping devices for other substances such as cannabis, which may impact findings. Finally, biochemical verification of switching was not conducted, potentially introducing uncertainty or bias into the outcome.

### Strengths

This study confirms previous findings that risk perceptions of ENDS compared to cigarettes are associated with subsequent switching among adult smokers who initiated with ENDS [31]. The current study extends these findings by focusing on adult smokers at the point of initiation with JUUL brand ENDS, allowing more complete capture of switching behavior in a large national sample of adult smokers. This study also examined comparative risk perceptions using different measures, which both indirectly and directly compared the perceived risk of JUUL to that of smoking. Although the focus on a single ENDS product may limit generalizability to other ENDS, it also limits variability in switch rates that may be due to varying performance of heterogenous ENDS products [55], rather than to risk perceptions, enabling a clearer read on the latter.

## Conclusions

Among established smokers who purchased and used JUUL ENDS, those who perceived JUUL to be less hazardous than smoking were more likely to switch away from smoking over the subsequent year. If this relationship is causal, it suggests that education to correct misperceptions about ENDS’ risk compared to cigarettes could encourage adult smokers to switch completely away from cigarettes. Further research should examine whether such education changes risk perceptions of ENDS and consequently increases switching rates. While all smokers, regardless of their level of smoking, would benefit from completely switching away from smoking, some of the health benefits scale steeply with cigarette consumption (e.g. cancer [56], in contrast to cardiovascular disease), so heavier smokers have the potential to experience an especially large benefit.

## Data Availability

Due to its proprietary nature, the analyzed data are not publicly available. Analytical code used to conduct the analyses is available upon a reasonable request to the corresponding author. The survey instrument for the current survey is publicly available at: https://www.juullabs.com/wp-content/uploads/2021/03/ADJUSST-Baseline-and-Follow-Up.pdf.

## Abbreviations

ADJUSST: Adult JUUL Switching and Smoking Trajectories
CPD: Cigarettes Per Day
ENDS: Electronic Nicotine Delivery System
GEE: Generalized Estimating Equations
